# Supplementary material for: The Impact on Autonomic Nervous System Activity during and Following Exercise in Adults: A Meta-Regression Study and Trial Sequential Analysis
Source: Medicina (Kaunas). 2024 Jul 28;60(8):1223. doi: 10.3390/medicina60081223 (PMC11356649; doi:10.3390/medicina60081223)
Supplement: Supplementary file 1 [file medicina-60-01223-s001.zip › medicina-3117854-supplementary.pdf]

DOWNS AND BLACK CHECKLIST

In the present version of the checklist we modified the scoring of item 27 that refers to the power of the study. Instead of rating according to an available range of study powers, we rated whether the study or not performed power calculation. Accordingly the maximum score for item 27 was 1 (a power analysis was conducted) instead of 5 and thus the highest possible score for the checklist was 28 (instead of 32). Downs and Black score ranges were given corresponding quality levels as previously reported(Hooper, Jutai, Strong, & Russell-Minda, 2008): excellent (26-28); good (20-25); fair (15-19); and poor (≤14). The reviewers' results were compared by an external reviewer and discrepancies were resolved in a consensus meeting. The checklist can evaluate both randomized controlled and non-controlled trials.

The Effects of Exercise on Autonomic Nervous System Activity in Adults: A Meta-Regression Study with Trial Sequential Analysis (n=10)				
**Article Author & Name:**	**Raimundo et al, 2021**			**Notes/Justification**
**Reporting**	**0**	**1**	**2**	
1. *Is the hypothesis/aim/objective of the study clearly described?*		Yes		
2. *Are the main outcomes to be measured clearly described in the Introduction or Methods section?*<br>If the main outcomes are first mentioned in the Results section, the question should be answered no.		Yes		
3. *Are the characteristics of the patients included in the study clearly described ?*<br>In cohort studies and trials, inclusion and/or exclusion criteria should be given. In case-control studies, a case-definition and the source for controls should be given.		Yes		Described clearly in Table 1
4. *Are the interventions of interest clearly described?*<br>Treatments and placebo (where relevant) that are to be compared should be clearly described.		Yes		
5. *Are the distributions of principal confounders in each group of subjects tobe compared clearly described?*<br>A list of principal confounders is provided.		yes		
6. *Are the main findings of the study clearly described?*<br>Simple outcome data (including denominators and numerators) should be reported for all major findings so that the reader can check the major analyses and conclusions. (This question does not cover statistical tests which are considered below).		Yes		
7. *Does the study provide estimates of the random variability in the data for the main outcomes?*<br>In non normally distributed data the inter-quartile range of results should be		Yes		

reported. In normally distributed data the standard error, standard deviation or confidence intervals should be reported. If the distribution of the data is not described, it must be assumed that the estimates used were appropriate and the question should be answered yes.				
8. *Have all important adverse events that may be a consequence of the intervention been reported?*<br>This should be answered yes if the study demonstrates that there was a comprehensive attempt to measure adverse events. (A list of possible adverse events is provided).	No			No reporting of any attempt to measure adverse events
9. *Have the characteristics of patients lost to follow-up been described?*<br>This should be answered yes where there were no losses to follow-up or where losses to follow-up were so small that findings would be unaffected by their inclusion. This should be answered no where a study does not report the number of patients lost to follow-up.		Yes		There were no losses to follow-up
10. *Have actual probability values been reported (e.g. 0.035 rather than <0.05) for the main outcomes except where the probability value is less than 0.001?*		Yes		
**External Validity**<br>All the following criteria attempt to address the representativeness of the findings of the study and whether they may be generalised to the population from which the study subjects were derived.				
11. *Were the subjects asked to participate in the study representative of the entire population from which they were recruited?*<br>The study must identify the source population for patients and describe how the patients were selected. Patients would be representative if they comprised the entire source population, an unselected sample of consecutive patients, or a random sample. Random sampling is only feasible where a list of all members of the relevant population exists. Where a study does not report the proportion of the source population from which the patients are derived, the question should be answered as unable to determine.	Unable to Determine			
12. *Were those subjects who were prepared to participate representative of the entire population from which they were recruited?*<br>The proportion of those asked who agreed should be stated. Validation that the sample was representative would include demonstrating that the distribution of the main confounding factors was the same in the study sample and the source population.	Unable to determine			
13. *Were the staff, places, and facilities where the patients were treated, representative of the treatment the majority of patients receive?*<br>For the question to be answered yes the study should demonstrate that the intervention was representative of that in use in the source population. The question should be answered no if, for example, the intervention was undertaken in a specialist centre unrepresentative of the hospitals most of the source population would attend.	Unable to determine			No information regarding the exercise.
**Internal validity – bias**				

14. *Was an attempt made to blind study subjects to the intervention they have received?*<br>For studies where the patients would have no way of knowing which intervention they received, this should be answered yes.	No			Comparison between exercises – unable to blind these allocations.
15. *Was an attempt made to blind those measuring the main outcomes of the intervention?*	No			

16. *If any of the results of the study were based on "data dredging", was this made clear?*<br>Any analyses that had not been planned at the outset of the study should be clearly indicated. If no retrospective unplanned subgroup analyses were reported, then answer yes.	No			
17. *In trials and cohort studies, do the analyses adjust for different lengths of follow-up of patients, or in case-control studies, is the time period between the intervention and outcome the same for cases and controls?*<br>Where follow-up was the same for all study patients the answer should yes. If different lengths of follow-up were adjusted for by, for example, survival analysis the answer should be yes. Studies where differences in follow-up are ignored should be answered no.		Yes		Same follow-up period
18. *Were the statistical tests used to assess the main outcomes appropriate?* The statistical techniques used must be appropriate to the data. For example nonparametric methods should be used for small sample sizes. Where little statistical analysis has been undertaken but where there is no evidence of bias, the question should be answered yes. If the distribution of the data (normal or not) is not described it must be assumed that the estimates used were appropriate and the question should be answered yes.		Yes		
19. *Was compliance with the intervention/s reliable?*<br>Where there was non compliance with the allocated treatment or where there was contamination of one group, the question should be answered no. For studies where the effect of any misclassification was likely to bias any association to the null, the question should be answered yes.		Yes		
20. *Were the main outcome measures used accurate (valid and reliable)?*<br>For studies where the outcome measures are clearly described, the question should be answered yes. For studies which refer to other work or that demonstrates the outcome measures are accurate, the question should be answered as yes.		Yes		
**Internal validity - confounding (selection bias)**				
21. *Were the patients in different intervention groups (trials and cohort studies) or were the cases and controls (case-control studies) recruited from the same population?*<br>For example, patients for all comparison groups should be selected from the same hospital. The question should be answered unable to determine for cohort and casecontrol studies where there is no information concerning the source of patients included in the study.		Yes		
22. *Were study subjects in different intervention groups (trials and cohort studies) or were the cases and controls (case-control studies) recruited over the same period of time?*<br>For a study which does not specify the time period over which patients were recruited, the question should be answered as unable to determine.		Yes		

23. *Were study subjects randomised to intervention groups?* Studies which state that subjects were randomized should be answered yes except where method of randomisation would not ensure random allocation. For example alternate allocation would score no because it is predictable.	No			
24. *Was the randomised intervention assignment concealed from both patients and health care staff until recruitment was complete and irrevocable?* All non-randomised studies should be answered no. If assignment was	No			

concealed from patients but not from staff, it should be answered no.				
25. *Was there adequate adjustment for confounding in the analyses from which the main findings were drawn?*<br>This question should be answered no for trials if: the main conclusions of the study were based on analyses of treatment rather than intention to treat; the distribution of known confounders in the different treatment groups was not described; or the distribution of known confounders differed between the treatment groups but was not taken into account in the analyses. In nonrandomized studies if the effect of the main confounders was not investigated or confounding was demonstrated but no adjustment was made in the final analyses the question should be answered as no.	Unable to determine			
26. *Were losses of patients to follow-up taken into account?*<br>If the numbers of patients lost to follow-up are not reported, the question should be answered as unable to determine. If the proportion lost to follow- up was too small to affect the main findings, the question should be answered yes.		Yes		Appears to be sample of convenience with no patients lost to follow-up
**Power**				
27. *Did the study have sufficient power to detect a clinically important effect where the probability value for a difference being due to chance is less than 5%?*<br>Sample sizes have been calculated to detect a difference of x% and y%.		Yes		No power analysis performed, however effects documented demonstrate adequate power.
**Total score: 17/28**				

**Article Author & Name:**	**Gauche et al, 2017**			**Notes/Justification**
**Reporting**	**0**	**1**	**2**	
1. *Is the hypothesis/aim/objective of the study clearly described?*		Yes		
2. *Are the main outcomes to be measured clearly described in the Introduction or Methods section?*<br>If the main outcomes are first mentioned in the Results section, the question should be answered no.		Yes		
3. *Are the characteristics of the patients included in the study clearly described?*<br>In cohort studies and trials, inclusion and/or exclusion criteria should be given. In case-control studies, a case-definition and the source for controls should be given.		Yes		Described clearly in Table 1
4. *Are the interventions of interest clearly described?*<br>Treatments and placebo (where relevant) that are to be compared should be clearly described.		Yes		
5. *Are the distributions of principal confounders in each group of subjects to be compared clearly described?*<br>A list of principal confounders is provided.		yes		

6. *Are the main findings of the study clearly described?* Simple outcome data (including denominators and numerators) should be reported for all major findings so that the reader can check the major analyses and conclusions. (This question does not cover statistical tests which are considered below).		Yes		Written in table 2 and table 3
7. *Does the study provide estimates of the random variability in the data for the main outcomes?* In non-normally distributed data the inter-quartile range of results should be		Yes		

reported. In normally distributed data the standard error, standard deviation or confidence intervals should be reported. If the distribution of the data is not described, it must be assumed that the estimates used were appropriate and the question should be answered yes.				
8. *Have all important adverse events that may be a consequence of the intervention been reported?*<br>This should be answered yes if the study demonstrates that there was a comprehensive attempt to measure adverse events. (A list of possible adverse events is provided).	No			No reporting of any attempt to measure adverse events
9. *Have the characteristics of patients lost to follow-up been described?*<br>This should be answered yes where there were no losses to follow-up or where losses to follow-up were so small that findings would be unaffected by their inclusion. This should be answered no where a study does not report the number of patients lost to follow-up.		Yes		There were no losses to follow-up
10. *Have actual probability values been reported (e.g. 0.035 rather than <0.05) for the main outcomes except where the probability value is less than 0.001?*		Yes		
**External Validity**<br>All the following criteria attempt to address the representativeness of the findings of the study and whether they may be generalised to the population from which the study subjects were derived.				
11. *Were the subjects asked to participate in the study representative of the entire population from which they were recruited?*<br>The study must identify the source population for patients and describe how the patients were selected. Patients would be representative if they comprised the entire source population, an unselected sample of consecutive patients, or a random sample. Random sampling is only feasible where a list of all members of the relevant population exists. Where a study does not report the proportion of the source population from which the patients are derived, the question should be answered as unable to determine.	Unable to determine			
12. *Were those subjects who were prepared to participate representative of the entire population from which they were recruited?*<br>The proportion of those asked who agreed should be stated. Validation that the sample was representative would include demonstrating that the distribution of the main confounding factors was the same in the study sample and the source population.	Unable to determine			
13. *Were the staff, places, and facilities where the patients were treated, representative of the treatment the majority of patients receive?*<br>For the question to be answered yes the study should demonstrate that the intervention was representative of that in use in the source population. The question should be answered no if, for example, the intervention was undertaken in a specialist centre unrepresentative of the hospitals most of the source population would attend.	Unable to determine			No information regarding the exercise.
**Internal validity – bias**				

14. *Was an attempt made to blind study subjects to the intervention they have received?*<br>For studies where the patients would have no way of knowing which intervention they received, this should be answered yes.	No			Comparison between exercises – unable to blind these allocations.
15. *Was an attempt made to blind those measuring the main outcomes of the intervention?*	No			

16. *If any of the results of the study were based on "data dredging", was this made clear?*<br>Any analyses that had not been planned at the outset of the study should be clearly indicated. If no retrospective unplanned subgroup analyses were reported, then answer yes.	No			
17. *In trials and cohort studies, do the analyses adjust for different lengths of follow-up of patients, or in case-control studies, is the time period between the intervention and outcome the same for cases and controls?*<br>Where follow-up was the same for all study patients the answer should yes. If different lengths of follow-up were adjusted for by, for example, survival analysis the answer should be yes. Studies where differences in follow-up are ignored should be answered no.		Yes		
18. *Were the statistical tests used to assess the main outcomes appropriate?* The statistical techniques used must be appropriate to the data. For example nonparametric methods should be used for small sample sizes. Where little statistical analysis has been undertaken but where there is no evidence of bias, the question should be answered yes. If the distribution of the data (normal or not) is not described it must be assumed that the estimates used were appropriate and the question should be answered yes.		Yes		
19. *Was compliance with the intervention/s reliable?*<br>Where there was non compliance with the allocated treatment or where there was contamination of one group, the question should be answered no. For studies where the effect of any misclassification was likely to bias any association to the null, the question should be answered yes.		Yes		
20. *Were the main outcome measures used accurate (valid and reliable)?*<br>For studies where the outcome measures are clearly described, the question should be answered yes. For studies which refer to other work or that demonstrates the outcome measures are accurate, the question should be answered as yes.		Yes		
**Internal validity - confounding (selection bias)**				
21. *Were the patients in different intervention groups (trials and cohort studies) or were the cases and controls (case-control studies) recruited from the same population?*<br>For example, patients for all comparison groups should be selected from the same hospital. The question should be answered unable to determine for cohort and casecontrol studies where there is no information concerning the source of patients included in the study.		Yes		No comparison groups (cohort study)
22. *Were study subjects in different intervention groups (trials and cohort studies) or were the cases and controls (case-control studies) recruited over the same period of time?*<br>For a study which does not specify the time period over which patients were recruited, the question should be answered as unable to determine.		Yes		

23. *Were study subjects randomised to intervention groups?*<br>Studies which state that subjects were randomized should be answered yes except where method of randomisation would not ensure random allocation. For example alternate allocation would score no because it is predictable.	No			
24. *Was the randomised intervention assignment concealed from both patients and health care staff until recruitment was complete and irrevocable?*<br>All non-randomised studies should be answered no. If assignment was	No			

concealed from patients but not from staff, it should be answered no.				
25. *Was there adequate adjustment for confounding in the analyses from which the main findings were drawn?*<br>This question should be answered no for trials if: the main conclusions of the study were based on analyses of treatment rather than intention to treat; the distribution of known confounders in the different treatment groups was not described; or the distribution of known confounders differed between the treatment groups but was not taken into account in the analyses. In nonrandomized studies if the effect of the main confounders was not investigated or confounding was demonstrated but no adjustment was made in the final analyses the question should be answered as no.	No			
26. *Were losses of patients to follow-up taken into account?*<br>If the numbers of patients lost to follow-up are not reported, the question should be answered as unable to determine. If the proportion lost to follow- up was too small to affect the main findings, the question should be answered yes.		Yes		Appears to be sample of convenience with no patients lost to follow-up
**Power**				
27. *Did the study have sufficient power to detect a clinically important effect where the probability value for a difference being due to chance is less than 5%?*<br>Sample sizes have been calculated to detect a difference of x% and y%.		Yes		No power analysis performed, however effects documented demonstrate adequate power.
**Total score: 17/28**				

**Article Author & Name:**	**Droguett et al, 2015**			**Notes/Justification**
**Reporting**	**0**	**1**	**2**	
1. *Is the hypothesis/aim/objective of the study clearly described?*		Yes		
2. *Are the main outcomes to be measured clearly described in the Introduction or Methods section?*<br>If the main outcomes are first mentioned in the Results section, the question should be answered no.		Yes		
3. *Are the characteristics of the patients included in the study clearly described?*<br>In cohort studies and trials, inclusion and/or exclusion criteria should be given. In case-control studies, a case-definition and the source for controls should be given.		Yes		Described clearly in Table 1
4. *Are the interventions of interest clearly described?*<br>Treatments and placebo (where relevant) that are to be compared should be clearly described.		Yes		
5. *Are the distributions of principal confounders in each group of subjects to be compared clearly described?*<br>A list of principal confounders is provided.		yes		

6. *Are the main findings of the study clearly described?*<br>Simple outcome data (including denominators and numerators) should be reported for all major findings so that the reader can check the major analyses and conclusions. (This question does not cover statistical tests which are considered below).		Yes		
7. *Does the study provide estimates of the random variability in the data for the main outcomes?*<br>In non normally distributed data the inter-quartile range of results should be reported. In normally distributed data the standard error, standard		Yes		

or confidence intervals should be reported. If the distribution of the data is not described, it must be assumed that the estimates used were appropriate and the question should be answered yes.				
8. *Have all important adverse events that may be a consequence of the intervention been reported?* This should be answered yes if the study demonstrates that there was a comprehensive attempt to measure adverse events. (A list of possible adverse events is provided).	No			No reporting of any attempt to measure adverse events
9. *Have the characteristics of patients lost to follow-up been described?* This should be answered yes where there were no losses to follow-up or where losses to follow-up were so small that findings would be unaffected by their inclusion. This should be answered no where a study does not report the number of patients lost to follow-up.		Yes		There were no losses to follow-up
10. *Have actual probability values been reported (e.g. 0.035 rather than <0.05) for the main outcomes except where the probability value is less than 0.001?*		Partially		
**External Validity** All the following criteria attempt to address the representativeness of the findings of the study and whether they may be generalised to the population from which the study subjects were derived.				
11. *Were the subjects asked to participate in the study representative of the entire population from which they were recruited?* The study must identify the source population for patients and describe how the patients were selected. Patients would be representative if they comprised the entire source population, an unselected sample of consecutive patients, or a random sample. Random sampling is only feasible where a list of all members of the relevant population exists. Where a study does not report the proportion of the source population from which the patients are derived, the question should be answered as unable to determine.	No			
12. *Were those subjects who were prepared to participate representative of the entire population from which they were recruited?* The proportion of those asked who agreed should be stated. Validation that the sample was representative would include demonstrating that the distribution of the main confounding factors was the same in the study sample and the source population.	Unable to determine			
13. *Were the staff, places, and facilities where the patients were treated, representative of the treatment the majority of patients receive?* For the question to be answered yes the study should demonstrate that the intervention was representative of that in use in the source population. The question should be answered no if, for example, the intervention was undertaken in a specialist centre unrepresentative of the hospitals most of the source population would attend.	Unable to determine			No information regarding the exercise.
**Internal validity – bias**				

14. *Was an attempt made to blind study subjects to the intervention they have received ?*<br>For studies where the patients would have no way of knowing which intervention they received, this should be answered yes.	No			Case control study, no attempt made to blind.
15. *Was an attempt made to blind those measuring the main outcomes of the intervention?*	No			
16. *If any of the results of the study were based on "data dredging", was this*	No			

made clear?<br>Any analyses that had not been planned at the outset of the study should be clearly indicated. If no retrospective unplanned subgroup analyses were reported, then answer yes.				
17. *In trials and cohort studies, do the analyses adjust for different lengths of follow-up of patients, or in case-control studies, is the time period between the intervention and outcome the same for cases and controls?*<br>Where follow-up was the same for all study patients the answer should yes. If different lengths of follow-up were adjusted for by, for example, survival analysis the answer should be yes. Studies where differences in follow-up are ignored should be answered no.		Yes		
18. *Were the statistical tests used to assess the main outcomes appropriate?* The statistical techniques used must be appropriate to the data. For example nonparametric methods should be used for small sample sizes. Where little statistical analysis has been undertaken but where there is no evidence of bias, the question should be answered yes. If the distribution of the data (normal or not) is not described it must be assumed that the estimates used were appropriate and the question should be answered yes.		Yes		
19. *Was compliance with the intervention/s reliable?*<br>Where there was non compliance with the allocated treatment or where there was contamination of one group, the question should be answered no. For studies where the effect of any misclassification was likely to bias any association to the null, the question should be answered yes.		Yes		
20. *Were the main outcome measures used accurate (valid and reliable)?*<br>For studies where the outcome measures are clearly described, the question should be answered yes. For studies which refer to other work or that demonstrates the outcome measures are accurate, the question should be answered as yes.		Yes		
**Internal validity - confounding (selection bias)**				
21. *Were the patients in different intervention groups (trials and cohort studies) or were the cases and controls (case-control studies) recruited from the same population?*<br>For example, patients for all comparison groups should be selected from the same hospital. The question should be answered unable to determine for cohort and casecontrol studies where there is no information concerning the source of patients included in the study.		Yes		
22. *Were study subjects in different intervention groups (trials and cohort studies) or were the cases and controls (case-control studies) recruited over the same period of time?*<br>For a study which does not specify the time period over which patients were recruited, the question should be answered as unable to determine.		Yes		
23. *Were study subjects randomised to intervention groups?*<br>Studies which state that subjects were randomized should be answered yes except where method of randomisation would not ensure random allocation. For example alternate allocation would score no because it is predictable.		yes		

24. *Was the randomised intervention assignment concealed from both patients and health care staff until recruitment was complete and irrevocable?*<br>All non-randomised studies should be answered no. If assignment was concealed from patients but not from staff, it should be answered no.	No			
25. *Was there adequate adjustment for confounding in the analyses from*	No			

which the main findings were drawn?<br>This question should be answered no for trials if: the main conclusions of the study were based on analyses of treatment rather than intention to treat; the distribution of known confounders in the different treatment groups was not described; or the distribution of known confounders differed between the treatment groups but was not taken into account in the analyses. In nonrandomized studies if the effect of the main confounders was not investigated or confounding was demonstrated but no adjustment was made in the final analyses the question should be answered as no.				
26. *Were losses of patients to follow-up taken into account?*<br>If the numbers of patients lost to follow-up are not reported, the question should be answered as unable to determine. If the proportion lost to follow- up was too small to affect the main findings, the question should be answered yes.		yes		Appears to be sample of convenience with no patients lost to follow-up
**Power**				
27. *Did the study have sufficient power to detect a clinically important effect where the probability value for a difference being due to chance is less than 5%?* Sample sizes have been calculated to detect a difference of x% and y%.		yes		No power analysis performed, however effects documented demonstrate adequate power.
**Total score: 17/28**				

**Article Author & Name:**	**Machado-Vidotti et al, 2014**			**Notes/Justification**
**Reporting**	**0**	**1**	**2**	
1. *Is the hypothesis/aim/objective of the study clearly described?*		Yes		
2. *Are the main outcomes to be measured clearly described in the Introduction or Methods section?*<br>If the main outcomes are first mentioned in the Results section, the question should be answered no.		Yes		
3. *Are the characteristics of the patients included in the study clearly described?*<br>In cohort studies and trials, inclusion and/or exclusion criteria should be given. In case-control studies, a case-definition and the source for controls should be given.		Yes		
4. *Are the interventions of interest clearly described?*<br>Treatments and placebo (where relevant) that are to be compared should be clearly described.		Yes		
5. *Are the distributions of principal confounders in each group of subjects to be compared clearly described?*<br>A list of principal confounders is provided.		yes		
6. *Are the main findings of the study clearly described?*<br>Simple outcome data (including denominators and numerators) should be reported for all major findings so that the reader can check the major analyses and conclusions. (This question does not cover statistical tests which are considered below).		Yes		

7. *Does the study provide estimates of the random variability in the data for the main outcomes?*<br>In non normally distributed data the inter-quartile range of results should be reported. In normally distributed data the standard error, standard deviation or confidence intervals should be reported. If the distribution of the data is not described, it must be assumed that the estimates used were appropriate		Yes		

and the question should be answered yes.				
8. *Have all important adverse events that may be a consequence of the intervention been reported?* This should be answered yes if the study demonstrates that there was a comprehensive attempt to measure adverse events. (A list of possible adverse events is provided).	No			No reporting of any attempt to measure adverse events
9. *Have the characteristics of patients lost to follow-up been described?* This should be answered yes where there were no losses to follow-up or where losses to follow-up were so small that findings would be unaffected by their inclusion. This should be answered no where a study does not report the number of patients lost to follow-up.		Yes		There were no losses to follow-up
10. *Have actual probability values been reported(e.g. 0.035 rather than <0.05) for the main outcomes except where the probability value is less than 0.001?*		partially		
**External Validity** All the following criteria attempt to address the representativeness of the findings of the study and whether they may be generalised to the population from which the study subjects were derived.				
11. *Were the subjects asked to participate in the study representative of the entire population from which they were recruited?* The study must identify the source population for patients and describe how the patients were selected. Patients would be representative if they comprised the entire source population, an unselected sample of consecutive patients, or a random sample. Random sampling is only feasible where a list of all members of the relevant population exists. Where a study does not report the proportion of the source population from which the patients are derived, the question should be answered as unable to determine.	No			
12. *Were those subjects who were prepared to participate representative of the entire population from which they were recruited?* The proportion of those asked who agreed should be stated. Validation that the sample was representative would include demonstrating that the distribution of the main confounding factors was the same in the study sample and the source population.	Unable to determine			
13. *Were the staff, places, and facilities where the patients were treated, representative of the treatment the majority of patients receive?* For the question to be answered yes the study should demonstrate that the intervention was representative of that in use in the source population. The question should be answered no if, for example, the intervention was undertaken in a specialist centre unrepresentative of the hospitals most of the source population would attend.	Unable to determine			No information regarding the exercise.
**Internal validity – bias**				
14. *Was an attempt made to blind study subjects to the intervention they have received?* For studies where the patients would have no way of knowing which intervention they received, this should be answered yes.	No			Participants not blinded to treatment assignment at any point in study

15. *Was an attempt made to blind those measuring the main outcomes of the intervention?*	No			
16. *If any of the results of the study were based on "data dredging", was this made clear?* Any analyses that had not been planned at the outset of the study should be	No			

clearly indicated. If no retrospective unplanned subgroup analyses were reported, then answer yes.				
17. *In trials and cohort studies, do the analyses adjust for different lengths of follow-up of patients, or in case-control studies, is the time period between the intervention and outcome the same for cases and controls?*<br>Where follow-up was the same for all study patients the answer should yes. If different lengths of follow-up were adjusted for by, for example, survival analysis the answer should be yes. Studies where differences in follow-up are ignored should be answered no.		Yes		
18. *Were the statistical tests used to assess the main outcomes appropriate?* The statistical techniques used must be appropriate to the data. For example nonparametric methods should be used for small sample sizes. Where little statistical analysis has been undertaken but where there is no evidence of bias, the question should be answered yes. If the distribution of the data (normal or not) is not described it must be assumed that the estimates used were appropriate and the question should be answered yes.		Yes		
19. *Was compliance with the intervention/s reliable?*<br>Where there was non compliance with the allocated treatment or where there was contamination of one group, the question should be answered no. For studies where the effect of any misclassification was likely to bias any association to the null, the question should be answered yes.		Yes		
20. *Were the main outcome measures used accurate (valid and reliable)?*<br>For studies where the outcome measures are clearly described, the question should be answered yes. For studies which refer to other work or that demonstrates the outcome measures are accurate, the question should be answered as yes.		Yes		
**Internal validity - confounding (selection bias)**				
21. *Were the patients in different intervention groups (trials and cohort studies) or were the cases and controls (case-control studies) recruited from the same population?*<br>For example, patients for all comparison groups should be selected from the same hospital. The question should be answered unable to determine for cohort and case control studies where there is no information concerning the source of patients included in the study.		Yes		
22. *Were study subjects in different intervention groups (trials and cohort studies) or were the cases and controls (case-control studies) recruited over the same period of time?*<br>For a study which does not specify the time period over which patients were recruited, the question should be answered as unable to determine.		Yes		
23. *Were study subjects randomised to intervention groups?*<br>Studies which state that subjects were randomized should be answered yes except where method of randomisation would not ensure random allocation. For example alternate allocation would score no because it is predictable.		Yes		

24. *Was the randomised intervention assignment concealed from both patients and health care staff until recruitment was complete and irrevocable?* All non-randomised studies should be answered no. If assignment was concealed from patients but not from staff, it should be answered no.	No			
25. *Was there adequate adjustment for confounding in the analyses from which the main findings were drawn?*	Unable to determine			Unable to determine compliance of those allocated to exercises.

This question should be answered no for trials if: the main conclusions of the study were based on analyses of treatment rather than intention to treat; the distribution of known confounders in the different treatment groups was not described; or the distribution of known confounders differed between the treatment groups but was not taken into account in the analyses. In nonrandomized studies if the effect of the main confounders was not investigated or confounding was demonstrated but no adjustment was made in the final analyses the question should be answered as no.				
26. *Were losses of patients to follow-up taken into account?* If the numbers of patients lost to follow-up are not reported, the question should be answered as unable to determine. If the proportion lost to follow- up was too small to affect the main findings, the question should be answered yes.		Yes		Appears to be sample of convenience with no patients lost to follow-up
**Power**				
27. *Did the study have sufficient power to detect a clinically important effect where the probability value for a difference being due to chance is less than 5%?* Sample sizes have been calculated to detect a difference of x% and y%.		Yes		No power analysis performed, however effects documented demonstrate adequate power.
**Total score: 17/28**				

**Article Author & Name:**	**Parks et al, 2022**			**Notes/Justification**
**Reporting**	**0**	**1**	**2**	
1. *Is the hypothesis/aim/objective of the study clearly described?*		Yes		
2. *Are the main outcomes to be measured clearly described in the Introduction or Methods section?* If the main outcomes are first mentioned in the Results section, the question should be answered no.		Yes		
3. *Are the characteristics of the patients included in the study clearly described?* In cohort studies and trials, inclusion and/or exclusion criteria should be given. In case-control studies, a case-definition and the source for controls should be given.		Yes		Described clearly in Table 1
4. *Are the interventions of interest clearly described?* Treatments and placebo (where relevant) that are to be compared should be clearly described.		Yes		
5. *Are the distributions of principal confounders in each group of subjects to be compared clearly described?* A list of principal confounders is provided.		yes		
6. *Are the main findings of the study clearly described?* Simple outcome data (including denominators and numerators) should be reported for all major findings so that the reader can check the major analyses and conclusions. (This question does not cover statistical tests which are considered below).		Yes		

7. *Does the study provide estimates of the random variability in the data for the main outcomes?*<br>In non normally distributed data the inter-quartile range of results should be reported. In normally distributed data the standard error, standard deviation or confidence intervals should be reported. If the distribution of the data is		Yes		Tests of normality not described

not described, it must be assumed that the estimates used were appropriate and the question should be answered yes.				
8. *Have all important adverse events that may be a consequence of the intervention been reported?*<br>This should be answered yes if the study demonstrates that there was a comprehensive attempt to measure adverse events. (A list of possible adverse events is provided).	No			No reporting of any attempt to measure adverse events
9. *Have the characteristics of patients lost to follow-up been described?* This should be answered yes where there were no losses to follow-up or<br>where losses to follow-up were so small that findings would be unaffected by their inclusion. This should be answered no where a study does not report the number of patients lost to follow-up.		Yes		There were no losses to follow-up
10. *Have actual probability values been reported(e.g. 0.035 rather than <0.05) for the main outcomes except where the probability value is less than 0.001?*		partially		
**External Validity**<br>All the following criteria attempt to address the representativeness of the findings of the study and whether they may be generalised to the population from which the study subjects were derived.				
11. *Were the subjects asked to participate in the study representative of the entire population from which they were recruited?*<br>The study must identify the source population for patients and describe how the patients were selected. Patients would be representative if they comprised the entire source population, an unselected sample of consecutive patients, or a random sample. Random sampling is only feasible where a list of all members of the relevant population exists. Where a study does not report the proportion of the source population from which the patients are derived, the question should be answered as unable to determine.	Unable to determine			
12. *Were those subjects who were prepared to participate representative of the entire population from which they were recruited?*<br>The proportion of those asked who agreed should be stated. Validation that the sample was representative would include demonstrating that the distribution of the main confounding factors was the same in the study sample and the source population.	Unable to determine			
13. *Were the staff, places, and facilities where the patients were treated, representative of the treatment the majority of patients receive?*<br>For the question to be answered yes the study should demonstrate that the intervention was representative of that in use in the source population. The question should be answered no if, for example, the intervention was undertaken in a specialist centre unrepresentative of the hospitals most of the source population would attend.	Unable to determine			No information regarding the exercise.
**Internal validity – bias**				

14. *Was an attempt made to blind study subjects to the intervention they have received ?*<br>For studies where the patients would have no way of knowing which intervention they received, this should be answered yes.	No			Comparison between exercises – unable to blind these allocations.
15. *Was an attempt made to blind those measuring the main outcomes of the intervention?*	No			
16. *If any of the results of the study were based on "data dredging", was this made clear?*	No			

Any analyses that had not been planned at the outset of the study should be clearly indicated. If no retrospective unplanned subgroup analyses were reported, then answer yes.				
17. *In trials and cohort studies, do the analyses adjust for different lengths of follow-up of patients, or in case-control studies, is the time period between the intervention and outcome the same for cases and controls ?*<br>Where follow-up was the same for all study patients the answer should yes. If different lengths of follow-up were adjusted for by, for example, survival analysis the answer should be yes. Studies where differences in follow-up are ignored should be answered no.		Yes		
18. *Were the statistical tests used to assess the main outcomes appropriate?* The statistical techniques used must be appropriate to the data. For example nonparametric methods should be used for small sample sizes. Where little statistical analysis has been undertaken but where there is no evidence of bias, the question should be answered yes. If the distribution of the data (normal or not) is not described it must be assumed that the estimates used were appropriate and the question should be answered yes.		Yes		
19. *Was compliance with the intervention/s reliable?*<br>Where there was non compliance with the allocated treatment or where there was contamination of one group, the question should be answered no. For studies where the effect of any misclassification was likely to bias any association to the null, the question should be answered yes.		Yes		
20. *Were the main outcome measures used accurate (valid and reliable)?*<br>For studies where the outcome measures are clearly described, the question should be answered yes. For studies which refer to other work or that demonstrates the outcome measures are accurate, the question should be answered as yes.		Yes		
**Internal validity - confounding (selection bias)**				
21. *Were the patients in different intervention groups (trials and cohort studies) or were the cases and controls (case-control studies) recruited from the same population?*<br>For example, patients for all comparison groups should be selected from the same hospital. The question should be answered unable to determine for cohort and casecontrol studies where there is no information concerning the source of patients included in the study.		Yes		
22. *Were study subjects in different intervention groups (trials and cohort studies) or were the cases and controls (case-control studies) recruited over the same period of time?*<br>For a study which does not specify the time period over which patients were recruited, the question should be answered as unable to determine.		Yes		
23. *Were study subjects randomised to intervention groups?*<br>Studies which state that subjects were randomized should be answered yes except where method of randomisation would not ensure random allocation. For example alternate allocation would score no because it is		Yes		

24. *Was the randomised intervention assignment concealed from both patients and health care staff until recruitment was complete and irrevocable?*<br>All non-randomised studies should be answered no. If assignment was concealed from patients but not from staff, it should be answered no.	No			
25. *Was there adequate adjustment for confounding in the analyses from which the main findings were drawn?*	No			

This question should be answered no for trials if: the main conclusions of the study were based on analyses of treatment rather than intention to treat; the distribution of known confounders in the different treatment groups was not described; or the distribution of known confounders differed between the treatment groups but was not taken into account in the analyses. In nonrandomized studies if the effect of the main confounders was not investigated or confounding was demonstrated but no adjustment was made in the final analyses the question should be answered as no.				
26. *Were losses of patients to follow-up taken into account?* If the numbers of patients lost to follow-up are not reported, the question should be answered as unable to determine. If the proportion lost to follow- up was too small to affect the main findings, the question should be answered yes.		Yes		Appears to be sample of convenience with no patients lost to follow-up
**Power**				
27. *Did the study have sufficient power to detect a clinically important effect where the probability value for a difference being due to chance is less than 5%?* Sample sizes have been calculated to detect a difference of x% and y%.		Yes		No power analysis performed, however effects documented demonstrate adequate power.
**Total score: 17/28**				

**Article Author & Name:**	**Sun et al, 2016**			**Notes/Justification**
**Reporting**	**0**	**1**	**2**	
1. *Is the hypothesis/aim/objective of the study clearly described?*		Yes		
2. *Are the main outcomes to be measured clearly described in the Introduction or Methods section?* If the main outcomes are first mentioned in the Results section, the question should be answered no.		Yes		
3. *Are the characteristics of the patients included in the study clearly described?* In cohort studies and trials, inclusion and/or exclusion criteria should be given. In case-control studies, a case-definition and the source for controls should be given.		Yes		Described clearly in Table 1
4. *Are the interventions of interest clearly described?* Treatments and placebo (where relevant) that are to be compared should be clearly described.		Yes		
5. *Are the distributions of principal confounders in each group of subjects to be compared clearly described?* A list of principal confounders is provided.		Yes		

6. *Are the main findings of the study clearly described?*<br>Simple outcome data (including denominators and numerators) should be reported for all major findings so that the reader can check the major analyses and conclusions. (This question does not cover statistical tests which are considered below).		Yes		
7. *Does the study provide estimates of the random variability in the data for the main outcomes?*<br>In non normally distributed data the inter-quartile range of results should be reported. In normally distributed data the standard error, standard deviation or confidence intervals should be reported. If the distribution of the data is not described, it must be assumed that the estimates used were appropriate and the question should be answered yes.		Yes		No test of normality described

8. *Have all important adverse events that may be a consequence of the intervention been reported?*<br>This should be answered yes if the study demonstrates that there was a comprehensive attempt to measure adverse events. (A list of possible adverse events is provided).	No			No reporting of any attempt to measure adverse events
9. *Have the characteristics of patients lost to follow-up been described?* This should be answered yes where there were no losses to follow-up or<br>where losses to follow-up were so small that findings would be unaffected by their inclusion. This should be answered no where a study does not report the number of patients lost to follow-up.		Yes		There were no losses to follow-up
10. *Have actual probability values been reported (e.g. 0.035 rather than <0.05) for the main outcomes except where the probability value is less than 0.001?*		partially		
**External Validity**<br>All the following criteria attempt to address the representativeness of the findings of the study and whether they may be generalised to the population from which the study subjects were derived.				
11. *Were the subjects asked to participate in the study representative of the entire population from which they were recruited?*<br>The study must identify the source population for patients and describe how the patients were selected. Patients would be representative if they comprised the entire source population, an unselected sample of consecutive patients, or a random sample. Random sampling is only feasible where a list of all members of the relevant population exists. Where a study does not report the proportion of the source population from which the patients are derived, the question should be answered as unable to determine.	Unable to determine			
12. *Were those subjects who were prepared to participate representative of the entire population from which they were recruited?*<br>The proportion of those asked who agreed should be stated. Validation that the sample was representative would include demonstrating that the distribution of the main confounding factors was the same in the study sample and the source population.	Unable to determine			
13. *Were the staff, places, and facilities where the patients were treated, representative of the treatment the majority of patients receive?*<br>For the question to be answered yes the study should demonstrate that the intervention was representative of that in use in the source population. The question should be answered no if, for example, the intervention was undertaken in a specialist centre unrepresentative of the hospitals most of the source population would attend.	Unable to determine			No information regarding the exercise.
**Internal validity – bias**				
14. *Was an attempt made to blind study subjects to the intervention they have received?*<br>For studies where the patients would have no way of knowing which intervention they received, this should be answered yes.	No			Comparison between exercises – unable to blind these allocations.
15. *Was an attempt made to blind those measuring the main outcomes of the intervention?*	No			

16. *If any of the results of the study were based on "data dredging", was this made clear?*<br>Any analyses that had not been planned at the outset of the study should be clearly indicated. If no retrospective unplanned subgroup analyses were	No			

reported, then answer yes.				
17. *In trials and cohort studies, do the analyses adjust for different lengths of follow-up of patients, or in case-control studies, is the time period between the intervention and outcome the same for cases and controls?* Where follow-up was the same for all study patients the answer should yes. If different lengths of follow-up were adjusted for by, for example, survival analysis the answer should be yes. Studies where differences in follow-up are ignored should be answered no.		Yes		
18. *Were the statistical tests used to assess the main outcomes appropriate?* The statistical techniques used must be appropriate to the data. For example nonparametric methods should be used for small sample sizes. Where little statistical analysis has been undertaken but where there is no evidence of bias, the question should be answered yes. If the distribution of the data (normal or not) is not described it must be assumed that the estimates used were appropriate and the question should be answered yes.		Yes		No tests of normality described
19. *Was compliance with the intervention/s reliable?* Where there was non compliance with the allocated treatment or where there was contamination of one group, the question should be answered no. For studies where the effect of any misclassification was likely to bias any association to the null, the question should be answered yes.		yes		
20. *Were the main outcome measures used accurate (valid and reliable)?* For studies where the outcome measures are clearly described, the question should be answered yes. For studies which refer to other work or that demonstrates the outcome measures are accurate, the question should be answered as yes.		Yes		
**Internal validity - confounding (selection bias)**				
21. *Were the patients in different intervention groups (trials and cohort studies) or were the cases and controls (case-control studies) recruited from the same population?* For example, patients for all comparison groups should be selected from the same hospital. The question should be answered unable to determine for cohort and casecontrol studies where there is no information concerning the source of patients included in the study.		Yes		
22. *Were study subjects in different intervention groups (trials and cohort studies) or were the cases and controls (case-control studies) recruited over the same period of time?* For a study which does not specify the time period over which patients were recruited, the question should be answered as unable to determine.		Yes		
23. *Were study subjects randomised to intervention groups?* Studies which state that subjects were randomized should be answered yes except where method of randomisation would not ensure random allocation. For example alternate allocation would score no because it is predictable.	No			

24. *Was the randomised intervention assignment concealed from both patients and health care staff until recruitment was complete and irrevocable?*<br>All non-randomised studies should be answered no. If assignment was concealed from patients but not from staff, it should be answered no.	No			
25. *Was there adequate adjustment for confounding in the analyses from which the main findings were drawn?*<br>This question should be answered no for trials if: the main conclusions of the study were based on analyses of treatment rather than intention to treat;	Unable to determine			

the distribution of known confounders in the different treatment groups was not described; or the distribution of known confounders differed between the treatment groups but was not taken into account in the analyses. In nonrandomized studies if the effect of the main confounders was not investigated or confounding was demonstrated but no adjustment was made in the final analyses the question should be answered as no.				
26. *Were losses of patients to follow-up taken into account?*<br>If the numbers of patients lost to follow-up are not reported, the question should be answered as unable to determine. If the proportion lost to follow- up was too small to affect the main findings, the question should be answered yes.		Yes		Appears to be sample of convenience with no patients lost to follow-up
**Power**				
27. *Did the study have sufficient power to detect a clinically important effect where the probability value for a difference being due to chance is less than 5%?*<br>Sample sizes have been calculated to detect a difference of x% and y%.		Yes		No power analysis performed, however effects documented demonstrate adequate power.
**Total score: 17/28**				

**Article Author & Name:**	**Marôco et al, 2024**			**Notes/Justification**
**Reporting**	**0**	**1**	**2**	
1. *Is the hypothesis/aim/objective of the study clearly described?*		Yes		
2. *Are the main outcomes to be measured clearly described in the Introduction or Methods section?*<br>If the main outcomes are first mentioned in the Results section, the question should be answered no.		Yes		
3. *Are the characteristics of the patients included in the study clearly described?*<br>In cohort studies and trials, inclusion and/or exclusion criteria should be given. In case-control studies, a case-definition and the source for controls should be given.		Yes		
4. *Are the interventions of interest clearly described?*<br>Treatments and placebo (where relevant) that are to be compared should be clearly described.		Yes		
5. *Are the distributions of principal confounders in each group of subjects to be compared clearly described?*<br>A list of principal confounders is provided.		Yes		
6. *Are the main findings of the study clearly described?*<br>Simple outcome data (including denominators and numerators) should be reported for all major findings so that the reader can check the major analyses and conclusions. (This question does not cover statistical tests which are considered below).		Yes		

7. *Does the study provide estimates of the random variability in the data for the main outcomes?*<br>In non normally distributed data the inter-quartile range of results should be		Yes		

reported. In normally distributed data the standard error, standard deviation or confidence intervals should be reported. If the distribution of the data is not described, it must be assumed that the estimates used were appropriate and the question should be answered yes.				
8. *Have all important adverse events that may be a consequence of the intervention been reported?*<br>This should be answered yes if the study demonstrates that there was a comprehensive attempt to measure adverse events. (A list of possible adverse events is provided).	No			No adverse effects discussed
9. *Have the characteristics of patients lost to follow-up been described?*<br>This should be answered yes where there were no losses to follow-up or where losses to follow-up were so small that findings would be unaffected by their inclusion. This should be answered no where a study does not report the number of patients lost to follow-up.		Yes		No participants lost to follow-up.
10. *Have actual probability values been reported(e.g. 0.035 rather than <0.05) for the main outcomes except where the probability value is less than 0.001?*		Partially		
**External Validity**<br>All the following criteria attempt to address the representativeness of the findings of the study and whether they may be generalised to the population from which the study subjects were derived.				
11. *Were the subjects asked to participate in the study representative of the entire population from which they were recruited?*<br>The study must identify the source population for patients and describe how the patients were selected. Patients would be representative if they comprised the entire source population, an unselected sample of consecutive patients, or a random sample. Random sampling is only feasible where a list of all members of the relevant population exists. Where a study does not report the proportion of the source population from which the patients are derived, the question should be answered as unable to determine.	Unable to Determine			
12. *Were those subjects who were prepared to participate representative of the entire population from which they were recruited?*<br>The proportion of those asked who agreed should be stated. Validation that the sample was representative would include demonstrating that the distribution of the main confounding factors was the same in the study sample and the source population.	Unable to Determine			
13. *Were the staff, places, and facilities where the patients were treated, representative of the treatment the majority of patients receive?*<br>For the question to be answered yes the study should demonstrate that the intervention was representative of that in use in the source population. The question should be answered no if, for example, the intervention was undertaken in a specialist centre unrepresentative of the hospitals most of the source population would attend.	Unable to Determine			No information regarding the exercise.
**Internal validity – bias**				

14. *Was an attempt made to blind study subjects to the intervention they have received?*<br>For studies where the patients would have no way of knowing which intervention they received, this should be answered yes.	No			Comparison between exercises – unable to blind these allocations.
15. *Was an attempt made to blind those measuring the main outcomes of the intervention?*	No			
16. *If any of the results of the study were based on "data dredging", was this*	No			

made clear?<br>Any analyses that had not been planned at the outset of the study should be clearly indicated. If no retrospective unplanned subgroup analyses were reported, then answer yes.				
17. *In trials and cohort studies, do the analyses adjust for different lengths of follow-up of patients, or in case-control studies, is the time period between the intervention and outcome the same for cases and controls ?*<br>Where follow-up was the same for all study patients the answer should yes. If different lengths of follow-up were adjusted for by, for example, survival analysis the answer should be yes. Studies where differences in follow-up are ignored should be answered no.		Yes		
18. *Were the statistical tests used to assess the main outcomes appropriate?* The statistical techniques used must be appropriate to the data. For example nonparametric methods should be used for small sample sizes. Where little statistical analysis has been undertaken but where there is no evidence of bias, the question should be answered yes. If the distribution of the data (normal or not) is not described it must be assumed that the estimates used were appropriate and the question should be answered yes.		Yes		
19. *Was compliance with the intervention/s reliable?*<br>Where there was non compliance with the allocated treatment or where there was contamination of one group, the question should be answered no. For studies where the effect of any misclassification was likely to bias any association to the null, the question should be answered yes.		Yes		
20. *Were the main outcome measures used accurate (valid and reliable)?*<br>For studies where the outcome measures are clearly described, the question should be answered yes. For studies which refer to other work or that demonstrates the outcome measures are accurate, the question should be answered as yes.		Yes		
**Internal validity - confounding (selection bias)**				
21. *Were the patients in different intervention groups (trials and cohort studies) or were the cases and controls (case-control studies) recruited from the same population?*<br>For example, patients for all comparison groups should be selected from the same hospital. The question should be answered unable to determine for cohort and casecontrol studies where there is no information concerning the source of patients included in the study.		Yes		
22. *Were study subjects in different intervention groups (trials and cohort studies) or were the cases and controls (case-control studies) recruited over the same period of time?*<br>For a study which does not specify the time period over which patients were recruited, the question should be answered as unable to determine.		Yes		
23. *Were study subjects randomised to intervention groups?*<br>Studies which state that subjects were randomized should be answered yes except where method of randomisation would not ensure random allocation. For example alternate allocation would score no because it is predictable.	No			no randomization

24. *Was the randomised intervention assignment concealed from both patients and health care staff until recruitment was complete and irrevocable?* All non-randomised studies should be answered no. If assignment was concealed from patients but not from staff, it should be answered no.	No			
25. *Was there adequate adjustment for confounding in the analyses from which the main findings were drawn?*	Unable to Determine			

This question should be answered no for trials if: the main conclusions of the study were based on analyses of treatment rather than intention to treat; the distribution of known confounders in the different treatment groups was not described; or the distribution of known confounders differed between the treatment groups but was not taken into account in the analyses. In nonrandomized studies if the effect of the main confounders was not investigated or confounding was demonstrated but no adjustment was made in the final analyses the question should be answered as no.				
26. *Were losses of patients to follow-up taken into account?* If the numbers of patients lost to follow-up are not reported, the question should be answered as unable to determine. If the proportion lost to follow- up was too small to affect the main findings, the question should be answered yes.		Yes		None lost to follow-up
**Power**				
27. *Did the study have sufficient power to detect a clinically important effect where the probability value for a difference being due to chance is less than 5%?* Sample sizes have been calculated to detect a difference of x% and y%.		Yes		No power analysis performed, however effects documented demonstrate adequate power.
**Total score: 17/28**				

**Article Author & Name:**	**Bhati et al, 2019**			**Notes/Justification**
**Reporting**	**0**	**1**	**2**	
1. *Is the hypothesis/aim/objective of the study clearly described?*		Yes		
2. *Are the main outcomes to be measured clearly described in the Introduction or Methods section?* If the main outcomes are first mentioned in the Results section, the question should be answered no.		Yes		
3. *Are the characteristics of the patients included in the study clearly described?* In cohort studies and trials, inclusion and/or exclusion criteria should be given. In case-control studies, a case-definition and the source for controls should be given.		Yes		Described clearly in Table 1
4. *Are the interventions of interest clearly described?* Treatments and placebo (where relevant) that are to be compared should be clearly described.		Yes		
5. *Are the distributions of principal confounders in each group of subjects to be compared clearly described?* A list of principal confounders is provided.		yes		

6. *Are the main findings of the study clearly described?*<br>Simple outcome data (including denominators and numerators) should be reported for all major findings so that the reader can check the major analyses and conclusions. (This question does not cover statistical tests which are considered below).		Yes		
7. *Does the study provide estimates of the random variability in the data for the main outcomes?*<br>In non normally distributed data the inter-quartile range of results should be		Yes		

reported. In normally distributed data the standard error, standard deviation or confidence intervals should be reported. If the distribution of the data is not described, it must be assumed that the estimates used were appropriate and the question should be answered yes.				
8. *Have all important adverse events that may be a consequence of the intervention been reported?*<br>This should be answered yes if the study demonstrates that there was a comprehensive attempt to measure adverse events. (A list of possible adverse events is provided).	no			No reporting of any attempt to measure adverse events
9. *Have the characteristics of patients lost to follow-up been described?*<br>This should be answered yes where there were no losses to follow-up or where losses to follow-up were so small that findings would be unaffected by their inclusion. This should be answered no where a study does not report the number of patients lost to follow-up.		yes		no losses to follow-up
10. *Have actual probability values been reported(e.g. 0.035 rather than <0.05) for the main outcomes except where the probability value is less than 0.001?*		partially		
**External Validity**<br>All the following criteria attempt to address the representativeness of the findings of the study and whether they may be generalised to the population from which the study subjects were derived.				
11. *Were the subjects asked to participate in the study representative of the entire population from which they were recruited?*<br>The study must identify the source population for patients and describe how the patients were selected. Patients would be representative if they comprised the entire source population, an unselected sample of consecutive patients, or a random sample. Random sampling is only feasible where a list of all members of the relevant population exists. Where a study does not report the proportion of the source population from which the patients are derived, the question should be answered as unable to determine.	Unable to determine			
12. *Were those subjects who were prepared to participate representative of the entire population from which they were recruited?*<br>The proportion of those asked who agreed should be stated. Validation that the sample was representative would include demonstrating that the distribution of the main confounding factors was the same in the study sample and the source population.	Unable to determine			
13. *Were the staff, places, and facilities where the patients were treated, representative of the treatment the majority of patients receive?*<br>For the question to be answered yes the study should demonstrate that the intervention was representative of that in use in the source population. The question should be answered no if, for example, the intervention was undertaken in a specialist centre unrepresentative of the hospitals most of the source population would attend.	Unable to determine			No information regarding the exercise.
**Internal validity – bias**				

14. *Was an attempt made to blind study subjects to the intervention they have received?*<br>For studies where the patients would have no way of knowing which intervention they received, this should be answered yes.	No			Comparison between exercises – unable to blind these allocations.
15. *Was an attempt made to blind those measuring the main outcomes of the intervention?*	No			
16. *If any of the results of the study were based on "data dredging", was this*	Unable to determine			

made clear?<br>Any analyses that had not been planned at the outset of the study should be clearly indicated. If no retrospective unplanned subgroup analyses were reported, then answer yes.				
17. *In trials and cohort studies, do the analyses adjust for different lengths of follow-up of patients, or in case-control studies, is the time period between the intervention and outcome the same for cases and controls?*<br>Where follow-up was the same for all study patients the answer should yes. If different lengths of follow-up were adjusted for by, for example, survival analysis the answer should be yes. Studies where differences in follow-up are ignored should be answered no.		Yes		
18. *Were the statistical tests used to assess the main outcomes appropriate?* The statistical techniques used must be appropriate to the data. For example nonparametric methods should be used for small sample sizes. Where little statistical analysis has been undertaken but where there is no evidence of bias, the question should be answered yes. If the distribution of the data (normal or not) is not described it must be assumed that the estimates used were appropriate and the question should be answered yes.		Yes		
19. *Was compliance with the intervention/s reliable?*<br>Where there was non compliance with the allocated treatment or where there was contamination of one group, the question should be answered no. For studies where the effect of any misclassification was likely to bias any association to the null, the question should be answered yes.		Yes		
20. *Were the main outcome measures used accurate (valid and reliable)?*<br>For studies where the outcome measures are clearly described, the question should be answered yes. For studies which refer to other work or that demonstrates the outcome measures are accurate, the question should be answered as yes.		Yes		
**Internal validity - confounding (selection bias)**				
21. *Were the patients in different intervention groups (trials and cohort studies) or were the cases and controls (case-control studies) recruited from the same population?*<br>For example, patients for all comparison groups should be selected from the same hospital. The question should be answered unable to determine for cohort and casecontrol studies where there is no information concerning the source of patients included in the study.		Yes		
22. *Were study subjects in different intervention groups (trials and cohort studies) or were the cases and controls (case-control studies) recruited over the same period of time?*<br>For a study which does not specify the time period over which patients were recruited, the question should be answered as unable to determine.		Yes		

23. *Were study subjects randomised to intervention groups?* Studies which state that subjects were randomized should be answered yes except where method of randomisation would not ensure random allocation. For example alternate allocation would score no because it is predictable.	No			no randomization
24. *Was the randomised intervention assignment concealed from both patients and health care staff until recruitment was complete and irrevocable?* All non-randomised studies should be answered no. If assignment was concealed from patients but not from staff, it should be answered no.	No			
25. *Was there adequate adjustment for confounding in the analyses from which the main findings were drawn?*	No			

This question should be answered no for trials if: the main conclusions of the study were based on analyses of treatment rather than intention to treat; the distribution of known confounders in the different treatment groups was not described; or the distribution of known confounders differed between the treatment groups but was not taken into account in the analyses. In nonrandomized studies if the effect of the main confounders was not investigated or confounding was demonstrated but no adjustment was made in the final analyses the question should be answered as no.				
26. *Were losses of patients to follow-up taken into account?* If the numbers of patients lost to follow-up are not reported, the question should be answered as unable to determine. If the proportion lost to follow- up was too small to affect the main findings, the question should be answered yes.		yes		Appears to be sample of convenience with no patients lost to follow-up
**Power**				
27. *Did the study have sufficient power to detect a clinically important effect where the probability value for a difference being due to chance is less than 5%?* Sample sizes have been calculated to detect a difference of x% and y%.		Yes		No power analysis performed, however effects documented demonstrate adequate power.
**Total score: 17/28**				

**Article Author & Name:**	**Colberg et al, 2014**			**Notes/Justification**
**Reporting**	**0**	**1**	**2**	
1. *Is the hypothesis/aim/objective of the study clearly described?*		Yes		
2. *Are the main outcomes to be measured clearly described in the Introduction or Methods section?* If the main outcomes are first mentioned in the Results section, the question should be answered no.		Yes		
3. *Are the characteristics of the patients included in the study clearly described?* In cohort studies and trials, inclusion and/or exclusion criteria should be given. In case-control studies, a case-definition and the source for controls should be given.		Yes		
4. *Are the interventions of interest clearly described?* Treatments and placebo (where relevant) that are to be compared should be clearly described.		Yes		
5. *Are the distributions of principal confounders in each group of subjects to be compared clearly described?* A list of principal confounders is provided.		yes		
6. *Are the main findings of the study clearly described?* Simple outcome data (including denominators and numerators) should be reported for all major findings so that the reader can check the major analyses and conclusions. (This question does not cover statistical tests which are considered below).		Yes		

7. *Does the study provide estimates of the random variability in the data for the main outcomes?*<br>In non normally distributed data the inter-quartile range of results should be reported. In normally distributed data the standard error, standard		Yes		

or confidence intervals should be reported. If the distribution of the data is not described, it must be assumed that the estimates used were appropriate and the question should be answered yes.				
8. *Have all important adverse events that may be a consequence of the intervention been reported?* This should be answered yes if the study demonstrates that there was a comprehensive attempt to measure adverse events. (A list of possible adverse events is provided).	No			No reporting of any attempt to measure adverse events
9. *Have the characteristics of patients lost to follow-up been described?* This should be answered yes where there were no losses to follow-up or where losses to follow-up were so small that findings would be unaffected by their inclusion. This should be answered no where a study does not report the number of patients lost to follow-up.		Yes		There were no losses to follow-up
10. *Have actual probability values been reported (e.g. 0.035 rather than <0.05) for the main outcomes except where the probability value is less than 0.001?*		Partially		
**External Validity** All the following criteria attempt to address the representativeness of the findings of the study and whether they may be generalised to the population from which the study subjects were derived.				
11. *Were the subjects asked to participate in the study representative of the entire population from which they were recruited?* The study must identify the source population for patients and describe how the patients were selected. Patients would be representative if they comprised the entire source population, an unselected sample of consecutive patients, or a random sample. Random sampling is only feasible where a list of all members of the relevant population exists. Where a study does not report the proportion of the source population from which the patients are derived, the question should be answered as unable to determine.	Unable to Determine			
12. *Were those subjects who were prepared to participate representative of the entire population from which they were recruited?* The proportion of those asked who agreed should be stated. Validation that the sample was representative would include demonstrating that the distribution of the main confounding factors was the same in the study sample and the source population.	Unable to determine			
13. *Were the staff, places, and facilities where the patients were treated, representative of the treatment the majority of patients receive?* For the question to be answered yes the study should demonstrate that the intervention was representative of that in use in the source population. The question should be answered no if, for example, the intervention was undertaken in a specialist centre unrepresentative of the hospitals most of the source population would attend.	Unable to determine			No information regarding the exercise.
**Internal validity – bias**				

14. *Was an attempt made to blind study subjects to the intervention they have received ?* For studies where the patients would have no way of knowing which intervention they received, this should be answered yes.	No			Case control study, no attempt made to blind.
15. *Was an attempt made to blind those measuring the main outcomes of the intervention?*	No			
16. *If any of the results of the study were based on "data dredging", was this*	No			

made clear?<br>Any analyses that had not been planned at the outset of the study should be clearly indicated. If no retrospective unplanned subgroup analyses were reported, then answer yes.				
17. *In trials and cohort studies, do the analyses adjust for different lengths of follow-up of patients, or in case-control studies, is the time period between the intervention and outcome the same for cases and controls?*<br>Where follow-up was the same for all study patients the answer should yes. If different lengths of follow-up were adjusted for by, for example, survival analysis the answer should be yes. Studies where differences in follow-up are ignored should be answered no.		Yes		
18. *Were the statistical tests used to assess the main outcomes appropriate?* The statistical techniques used must be appropriate to the data. For example nonparametric methods should be used for small sample sizes. Where little statistical analysis has been undertaken but where there is no evidence of bias, the question should be answered yes. If the distribution of the data (normal or not) is not described it must be assumed that the estimates used were appropriate and the question should be answered yes.		Yes		
19. *Was compliance with the intervention/s reliable?*<br>Where there was non compliance with the allocated treatment or where there was contamination of one group, the question should be answered no. For studies where the effect of any misclassification was likely to bias any association to the null, the question should be answered yes.		Yes		
20. *Were the main outcome measures used accurate (valid and reliable)?*<br>For studies where the outcome measures are clearly described, the question should be answered yes. For studies which refer to other work or that demonstrates the outcome measures are accurate, the question should be answered as yes.		Yes		
**Internal validity - confounding (selection bias)**				
21. *Were the patients in different intervention groups (trials and cohort studies) or were the cases and controls (case-control studies) recruited from the same population?*<br>For example, patients for all comparison groups should be selected from the same hospital. The question should be answered unable to determine for cohort and casecontrol studies where there is no information concerning the source of patients included in the study.		Yes		
22. *Were study subjects in different intervention groups (trials and cohort studies) or were the cases and controls (case-control studies) recruited over the same period of time?*<br>For a study which does not specify the time period over which patients were recruited, the question should be answered as unable to determine.		Yes		
23. *Were study subjects randomised to intervention groups?*<br>Studies which state that subjects were randomized should be answered yes except where method of randomisation would not ensure random allocation. For example alternate allocation would score no because it is predictable.	No			

24. *Was the randomised intervention assignment concealed from both patients and health care staff until recruitment was complete and irrevocable?*<br>All non-randomised studies should be answered no. If assignment was concealed from patients but not from staff, it should be answered no.	No			
25. *Was there adequate adjustment for confounding in the analyses from*	Unable to determine			

which the main findings were drawn?<br>This question should be answered no for trials if: the main conclusions of the study were based on analyses of treatment rather than intention to treat; the distribution of known confounders in the different treatment groups was not described; or the distribution of known confounders differed between the treatment groups but was not taken into account in the analyses. In nonrandomized studies if the effect of the main confounders was not investigated or confounding was demonstrated but no adjustment was made in the final analyses the question should be answered as no.				
26. *Were losses of patients to follow-up taken into account?*<br>If the numbers of patients lost to follow-up are not reported, the question should be answered as unable to determine. If the proportion lost to follow- up was too small to affect the main findings, the question should be answered yes.		yes		Appears to be sample of convenience with no patients lost to follow-up
**Power**				
27. *Did the study have sufficient power to detect a clinically important effect where the probability value for a difference being due to chance is less than 5%?* Sample sizes have been calculated to detect a difference of x% and y%.		yes		No power analysis performed, however effects documented demonstrate adequate power.
**Total score: 17/28**				

**Article Author & Name:**	**Grieco et al, 2014**			**Notes/Justification**
**Reporting**	**0**	**1**	**2**	
1. *Is the hypothesis/aim/objective of the study clearly described?*		Yes		
2. *Are the main outcomes to be measured clearly described in the Introduction or Methods section?*<br>If the main outcomes are first mentioned in the Results section, the question		Yes		
3. *Are the characteristics of the patients included in the study clearly described?*<br>In cohort studies and trials, inclusion and/or exclusion criteria should be given. In case-control studies, a case-definition and the source for controls should be given.		Yes		Described clearly in Table 1
4. *Are the interventions of interest clearly described?*<br>Treatments and placebo (where relevant) that are to be compared should be<br>clearly described.		Yes		
5. *Are the distributions of principal confounders in each group of subjects to be compared clearly described?*<br>A list of principal confounders is provided.		yes		

6. *Are the main findings of the study clearly described?* Simple outcome data (including denominators and numerators) should be reported for all major findings so that the reader can check the major analyses and conclusions. (This question does not cover statistical tests which are considered below).		Yes		
7. *Does the study provide estimates of the random variability in the data for the main outcomes?* In non normally distributed data the inter-quartile range of results should be reported. In normally distributed data the standard error, standard		Yes		

or confidence intervals should be reported. If the distribution of the data is not described, it must be assumed that the estimates used were appropriate and the question should be answered yes.				
8. *Have all important adverse events that may be a consequence of the intervention been reported?* This should be answered yes if the study demonstrates that there was a comprehensive attempt to measure adverse events. (A list of possible adverse events is provided).	No			No reporting of any attempt to measure adverse events
9. *Have the characteristics of patients lost to follow-up been described?* This should be answered yes where there were no losses to follow-up or where losses to follow-up were so small that findings would be unaffected by their inclusion. This should be answered no where a study does not report the number of patients lost to follow-up.		Yes		There were no losses to follow-up
10. *Have actual probability values been reported (e.g. 0.035 rather than <0.05) for the main outcomes except where the probability value is less than 0.001?*		Partially		
**External Validity** All the following criteria attempt to address the representativeness of the findings of the study and whether they may be generalised to the population from which the study subjects were derived.				
11. *Were the subjects asked to participate in the study representative of the entire population from which they were recruited?* The study must identify the source population for patients and describe how the patients were selected. Patients would be representative if they comprised the entire source population, an unselected sample of consecutive patients, or a random sample. Random sampling is only feasible where a list of all members of the relevant population exists. Where a study does not report the proportion of the source population from which the patients are derived, the question should be answered as unable to determine.	Unable to determine			
12. *Were those subjects who were prepared to participate representative of the entire population from which they were recruited?* The proportion of those asked who agreed should be stated. Validation that the sample was representative would include demonstrating that the distribution of the main confounding factors was the same in the study sample and the source population.	Unable to determine			
13. *Were the staff, places, and facilities where the patients were treated, representative of the treatment the majority of patients receive?* For the question to be answered yes the study should demonstrate that the intervention was representative of that in use in the source population. The question should be answered no if, for example, the intervention was undertaken in a specialist centre unrepresentative of the hospitals most of the source population would attend.	Unable to determine			No information regarding the exercise.
**Internal validity – bias**				

14. *Was an attempt made to blind study subjects to the intervention they have received ?*<br>For studies where the patients would have no way of knowing which intervention they received, this should be answered yes.	No			Case control study, no attempt made to blind.
15. *Was an attempt made to blind those measuring the main outcomes of the intervention?*	No			
16. *If any of the results of the study were based on "data dredging", was this*	No			

made clear? Any analyses that had not been planned at the outset of the study should be clearly indicated. If no retrospective unplanned subgroup analyses were reported, then answer yes.				
17. *In trials and cohort studies, do the analyses adjust for different lengths of follow-up of patients, or in case-control studies, is the time period between the intervention and outcome the same for cases and controls?* Where follow-up was the same for all study patients the answer should yes. If different lengths of follow-up were adjusted for by, for example, survival analysis the answer should be yes. Studies where differences in follow-up are ignored should be answered no.		Yes		
18. *Were the statistical tests used to assess the main outcomes appropriate?* The statistical techniques used must be appropriate to the data. For example nonparametric methods should be used for small sample sizes. Where little statistical analysis has been undertaken but where there is no evidence of bias, the question should be answered yes. If the distribution of the data (normal or not) is not described it must be assumed that the estimates used were appropriate and the question should be answered yes.		Yes		
19. *Was compliance with the intervention/s reliable?* Where there was non compliance with the allocated treatment or where there was contamination of one group, the question should be answered no. For studies where the effect of any misclassification was likely to bias any association to the null, the question should be answered yes.		Yes		
20. *Were the main outcome measures used accurate (valid and reliable)?* For studies where the outcome measures are clearly described, the question should be answered yes. For studies which refer to other work or that demonstrates the outcome measures are accurate, the question should be answered as yes.		Yes		
**Internal validity - confounding (selection bias)**				
21. *Were the patients in different intervention groups (trials and cohort studies) or were the cases and controls (case-control studies) recruited from the same population?* For example, patients for all comparison groups should be selected from the same hospital. The question should be answered unable to determine for cohort and casecontrol studies where there is no information concerning the source of patients included in the study.		Yes		
22. *Were study subjects in different intervention groups (trials and cohort studies) or were the cases and controls (case-control studies) recruited over the same period of time?* For a study which does not specify the time period over which patients were recruited, the question should be answered as unable to determine.		Yes		

23. *Were study subjects randomised to intervention groups?*<br>Studies which state that subjects were randomized should be answered yes except where method of randomisation would not ensure random allocation. For example alternate allocation would score no because it is predictable.		yes		
24. *Was the randomised intervention assignment concealed from both patients and health care staff until recruitment was complete and irrevocable?*<br>All non-randomised studies should be answered no. If assignment was concealed from patients but not from staff, it should be answered no.	No			
25. *Was there adequate adjustment for confounding in the analyses from*	No			

which the main findings were drawn? This question should be answered no for trials if: the main conclusions of the study were based on analyses of treatment rather than intention to treat; the distribution of known confounders in the different treatment groups was not described; or the distribution of known confounders differed between the treatment groups but was not taken into account in the analyses. In nonrandomized studies if the effect of the main confounders was not investigated or confounding was demonstrated but no adjustment was made in the final analyses the question should be answered as no.				
26. *Were losses of patients to follow-up taken into account?* If the numbers of patients lost to follow-up are not reported, the question should be answered as unable to determine. If the proportion lost to follow- up was too small to affect the main findings, the question should be answered yes.		yes		Appears to be sample of convenience with no patients lost to follow-up
**Power**				
27. *Did the study have sufficient power to detect a clinically important effect where the probability value for a difference being due to chance is less than 5%?* Sample sizes have been calculated to detect a difference of x% and y%.		yes		No power analysis performed, however effects documented demonstrate adequate power.
**Total score: 18/28**				
